# Distinct regions of the intrinsically disordered protein MUT-16 mediate assembly of a small RNA amplification complex and promote phase separation of *Mutator* foci

**DOI:** 10.1371/journal.pgen.1007542

**Published:** 2018-07-23

**Authors:** Celja J. Uebel, Dorian C. Anderson, Lisa M. Mandarino, Kevin I. Manage, Stephan Aynaszyan, Carolyn M. Phillips

**Affiliations:** Department of Biological Sciences, University of Southern California, Los Angeles, California, United States of America; University of Cambridge, UNITED KINGDOM

## Abstract

In *C*. *elegans*, efficient RNA silencing requires small RNA amplification mediated by RNA-dependent RNA polymerases (RdRPs). RRF-1, an RdRP, and other *Mutator* complex proteins localize to *Mutator* foci, which are perinuclear germline foci that associate with nuclear pores and P granules to facilitate small RNA amplification. The *Mutator* complex protein MUT-16 is critical for *Mutator* foci assembly. By analyzing small deletions of MUT-16, we identify specific regions of the protein that recruit other *Mutator* complex components and demonstrate that it acts as a scaffolding protein. We further determine that the C-terminal region of MUT-16, a portion of which contains predicted intrinsic disorder, is necessary and sufficient to promote *Mutator* foci formation. Finally, we establish that MUT-16 foci have many properties consistent with a phase-separated condensate and propose that *Mutator* foci form through liquid-liquid phase separation of MUT-16. P granules, which contain additional RNA silencing proteins, have previously been shown to have liquid-like properties. Thus, RNA silencing in *C*. *elegans* germ cells may rely on multiple phase-separated compartments through which sorting, processing, and silencing of mRNAs occurs.

## Author summary

Small RNAs are a driving force behind the regulation of both essential genes and deleterious transcripts. The *Mutator* complex is critical to the amplification of high levels of small RNAs and it requires the protein MUT-16 for its assembly. Here we investigate the function of MUT-16 by generating small deletions in the *mut-16* gene. Through analysis of the subsequently altered protein, we demonstrate that MUT-16 functions as a scaffold, bringing together many other proteins required for small RNA biogenesis and amplification. Furthermore, we identified a fragment of MUT-16 that is sufficient to promote assembly of MUT-16 into foci that are dynamic and responsive to environmental conditions. We propose that these *Mutator* foci behave like liquid droplets within the cell, similar to the immiscibility of oil droplets in water. *Mutator* foci localize to the periphery of germ cell nuclei near P granules, which also have liquid-like properties and contain many factors involved in RNA silencing. Thus, our data suggest that RNA silencing is mediated by compartments of RNAs and proteins in liquid-like assemblies at the periphery of germ cell nuclei.

## Introduction

RNA silencing is an anciently conserved pathway that regulates gene expression in most eukaryotes. Key to this pathway are members of the Argonaute protein family, which bind a diverse set of small regulatory RNAs, ranging from ~18–30 nucleotides in length. This small RNA-Argonaute complex regulates fully or partially complementary mRNAs at the level of transcription, translation, and stability [1,2]. By regulating both endogenous and foreign RNAs, small RNAs maintain proper gene expression, silence deleterious RNA products, and play critical roles in development, chromosome segregation, transposon silencing, fertility, and viral defense [2,3].

Small RNAs can be generated through a variety of mechanisms. Long double-stranded RNAs (dsRNAs), which can be produced from exogenous or endogenous sources, are cleaved into small-interfering RNAs (siRNAs) by the RNase III-like enzyme Dicer [4,5]. When these siRNAs are produced from a primary dsRNA source such as viruses or convergent transcription, they are referred to as primary siRNAs. In many organisms, including *C*. *elegans*, plants, and fungi, RNA-dependent RNA polymerases (RdRPs) use mRNAs targeted by primary siRNAs as a template to amplify and maintain the silencing signal [6–9]. RdRPs can function either by synthesis of additional long dsRNA and coordinated cleavage by Dicer into secondary siRNAs, or, as in *C*. *elegans*, RdRPs can directly synthesize secondary siRNAs antisense to the target mRNA [10–13].

In previous work, we characterized MUT-16 as a protein essential for RNA silencing and nucleation of a nuclear pore-associated RNA silencing compartment in *C*. *elegans* germ cells called *Mutator* foci [14]. MUT-16 recruits many other proteins required for RNA interference and endogenous small RNA biogenesis to *Mutator* foci, including the nucleotidyl transferase MUT-2, the 3’-5’ exonuclease MUT-7, the DEAD-box RNA helicases MUT-14 and SMUT-1, and two proteins of unknown function, RDE-2 and MUT-15 [14,15]. Additionally, the RNA-dependent RNA polymerase RRF-1 and the Zc3h12a-like ribonuclease RDE-8 localize to *Mutator* foci, however their dependencies for localization were not known [14,16]. No proteins have yet been identified that are required for MUT-16 localization, suggesting that MUT-16 may be the primary mediator of *Mutator* foci formation.

*Mutator* foci are considered hubs of siRNA amplification because mutations in *mut-16* or any of the associated *Mutator* complex proteins (*mut-2*, *mut-7*, *mut-14 smut-1*, *mut-15*, *rde-2*, *rde-8*, or *rrf-1*) result in a substantial loss of the RdRP-dependent secondary siRNAs [7,9,14–17]. Furthermore, *Mutator* foci reside adjacent to another ribonucleoprotein (RNP) granule, the P granule, which contains additional proteins associated with RNA silencing and mRNA decay [14]. Notably, the Argonaute protein WAGO-1, which binds secondary siRNAs synthesized in the *Mutator* complex, localizes to and presumably silences mRNAs complementary to its bound siRNAs in the P granule [7,18]. It is unclear how mRNAs targeted by primary Argonaute proteins get trafficked to the *Mutator* foci or how siRNAs generated in the *Mutator* foci end up bound by WAGO-1 in the P granule. However, the close juxtaposition between the P granule, the *Mutator* foci, and the nuclear pore suggests a role for sorting RNAs and proteins between compartments during the multi-step process of mRNA recognition and RNA silencing.

Little is known about how MUT-16 nucleates *Mutator* foci, but one clue comes from its protein sequence. MUT-16 is highly enriched in disorder-promoting amino acids such as glutamine and proline [14,19]. Intrinsically disordered regions (IDRs), which lack predicted structure, are often found in proteins linked to the formation of RNA granules (i.e. the nucleolus, P bodies, stress granules, and germ granules) [20–27]. Transient interactions between IDR-containing proteins can be a driving force for RNA granule formation through protein condensation and liquid-liquid phase separation, which occurs when proteins and RNAs self-organize to form a distinct compartment with liquid-like characteristics, but separate from the cytoplasm or nucleoplasm. This liquid droplet or granule, sometimes referred to as membrane-less organelle, can readily exchange components with the surrounding cytoplasm or nucleoplasm [21,24,26,28]. Thus, liquid-liquid phase separation can facilitate reactions by increasing the local concentration of proteins or RNA, as has been proposed for the nucleolus [26]. Alternatively, condensation can segregate certain factors away from the cytoplasm, for example sequestration of mRNAs and translational repressors into P bodies [29].

Here we demonstrate that the assembly of the *Mutator* complex and the formation of *Mutator* foci are mediated by the intrinsically disordered protein MUT-16. MUT-16 acts as a scaffold to recruit RRF-1 and other *Mutator* complex proteins, and it promotes foci formation through its C-terminal region. We further show that *Mutator* foci depend on weak hydrophobic interactions for their formation and form in a concentration and temperature-dependent manner. *Mutator* foci also recover rapidly after photobleaching. Thus, our data suggest that *Mutator* foci are phase-separated compartments with liquid-like properties that associate with P granules and nuclear pores in the cytoplasm of germ cells to promote RNA silencing.

## Results

### MUT-16 and orthologs contain predicted intrinsically disordered regions

**Fig 1:**
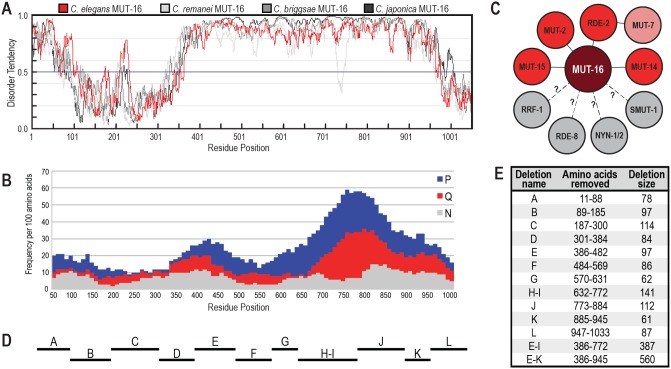
MUT-16 and orthologs contain a high degree of predicted disorder. (A) Graph comparing disorder tendency of *C*. *elegans* MUT-16 (isoform b, WP:CE40347) in red to orthologs in *C*. *remanei* (RP:RP48608) in light gray, *C*. *briggsae* (BP:CBP44329) in medium gray, and *C*. *japonica* (JA:JA63728) in dark gray using IUPRED and long disorder parameters (http://iupred.enzim.hu/). Scores above 0.5 indicate disorder. Residue positions correspond to the *C*. *elegans* MUT-16 sequence, but all proteins are similar in length. (B) Q, N, and P residues in *C*. *elegans* MUT-16 were counted in amino acid 100-mers, starting at position one, shifting 10 residues at a time, and displayed as stacked columns. Indicated residue positions are the mid-point of the 100-mer. (C) Schematic of known dependencies of *Mutator* foci formation. The requirements for localization of RRF-1, RDE-8, NYN-1, NYN-2, and SMUT-1 were not known prior to this work. (D, E) Diagram (D) and table (E) of MUT-16 deletions generated by CRISPR. Bars in (D) are drawn to scale relative to residue positions in (A,B).

In previous work, we observed that MUT-16 was both Q/N-rich and P-rich, predominantly in its C-terminal region and contains no other conserved domains [14]. Because amino acid sequences containing low complexity regions, such as Q/N-rich or P-rich regions, are often associated with disorder, we sought to identify regions of intrinsic disorder within the MUT-16 protein sequence using IUPred [30,31]. MUT-16 has a short IDR near the N-terminus (first ~100 amino acids) and a much longer IDR comprising approximately 60% of the protein (Fig 1A). In total, more than 70% of the protein is predicted to be unstructured. To determine if the intrinsically disordered nature of MUT-16 is a conserved feature of this protein, we used IUPred to predict IDR within the orthologs of MUT-16, in *C*. *remanei*, *C*. *briggsae*, and *C*. *japonica*. Despite the relatively low sequence conservation between orthologs, particularly in the C-terminal half of the protein [14], the conservation of IDRs was striking (Fig 1A). Like in *C*. *elegans*, the *C*. *remanei*, *C*. *briggsae*, and *C*. *japonica* MUT-16 proteins have a short IDR near the N-terminus and much longer IDR comprising the majority of the middle to C-terminal portions of the proteins.

To determine if the high incidence of Q/N/P residues is distributed throughout the IDR of MUT-16 or if it is restricted to a subset of the IDR, we counted the number of each of these residues in a sliding window of 100 amino acids, starting at position one and shifting 10 residues at a time. We observed a prominent peak of glutamine which overlapped with and was somewhat preceded by a prominent peak of proline (Fig 1B). These peaks spanned only a subset of the C-terminal IDR. Like the conservation of IDR, the pattern of enrichment of Q/N/P residues across MUT-16 orthologs in *C*. *remanei*, *C*. *briggsae*, and *C*. *japonica* is strikingly similar and suggestive of a functional role for Q/N/P residues (S1 Fig).

### Small deletions in *mut-16* are RNAi defective

Because IDRs have been shown to promote phase separation of proteins into liquid-like droplets [20–27], and MUT-16 forms foci *in vivo*, we sought to establish whether some or all of the MUT-16 IDR was required for *Mutator* foci formation. We also sought to identify regions of MUT-16 that directly recruit other *Mutator* complex proteins (Fig 1C). To this end, we generated a series of small deletions of the MUT-16 protein using CRISPR genome editing (Fig 1D and 1E). Each deletion removes between 61 and 141 residues (6–13%) of MUT-16. For simplicity, we refer to the deletions as ΔA through ΔL. We initially planned to make twelve deletions but due to technical constraints, the ΔH and ΔI deletions were combined to make the single, slightly larger ΔH-I deletion. We additionally generated two large deletions that remove most or all of the large IDR of MUT-16, which we will refer to as ΔE-I or ΔE-K.

**Fig 2:**
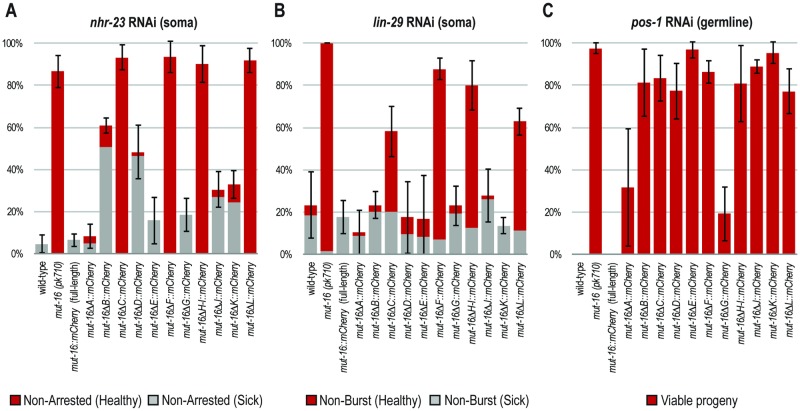
Susceptibility of *mut-16* deletion worms to somatic and germline RNAi. (A-C) *mut-16* deletion worms were assayed for their ability to respond to somatic (*nhr-23* or *lin-29*) or germline (*pos-1*) RNAi. For *nhr-23* RNAi (A) and *lin-29* RNAi (B), P0 animals were scored as having intermediate RNAi defects (gray bars), or fully penetrant RNAi defects (red bars). For *pos-1* RNAi (C), the F1 eggs and hatched larvae were counted to calculate % viable progeny from treated P0 animals. Weighted means and standard deviations were calculated from three independent RNAi trials of n = ~20 for *nhr-23* and *lin-29*, and n = ~90 F1 eggs from 4 P0 adults for *pos-1*.

A strain containing *mut-16(pk710)*, a null mutation in the *mut-16* gene, is defective in germline and somatic exogenous RNAi [14,17,32]. To determine the severity of RNAi defects caused by each *mut-16* deletion, we tested the response of each mutant strain to dsRNA targeting the germline gene *pos-1*, which causes embryonic lethality. We also tested their response to the somatic genes *nhr-23*, which causes larval arrest, and *lin-29*, which causes the intestine and gonad of the animal to rupture through the vulva at the larval to adult transition. Surprisingly, our RNAi assays revealed that only *mut-16* deletions ΔC, ΔF, ΔH-I, and ΔL have a significant impact on somatic RNAi (Fig 2A and 2B). The remaining deletions had only mild or no RNAi defect. In contrast, all deletions in *mut-16* had defects in germline RNAi, though deletions in ΔA and ΔG had more modest effects (Fig 2C). These data reveal that the majority of the MUT-16 protein is necessary for robust germline RNAi. In contrast, some regions of MUT-16 are dispensable for the response to at least some somatic RNAi clones, suggesting that the soma may be more resilient to mild perturbations in MUT-16.

### The C-terminal region of MUT-16 is critical for *Mutator* foci formation

**Fig 3:**
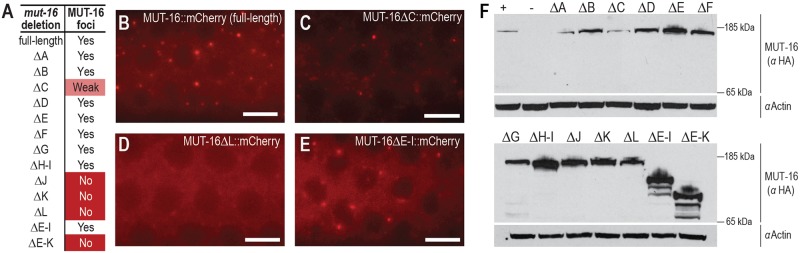
The C-terminal region of MUT-16 is necessary for *Mutator* foci formation. (A) Table indicates whether MUT-16 foci are present or absent in each *mut-16* deletion strain. Yes indicates foci present in the majority of animals, No indicates foci absent or severely disrupted, and Weak (for ΔC) indicates an intermediate phenotype where the fluorescence intensity of cytoplasmic MUT-16 appeared reduced relative to the other deletion lines. (B-E) Live imaging of MUT-16::mCherry expression and localization for control strain (B) or when ΔC (C), ΔL (D), or ΔE-I (E) deletions have been introduced into the *mut-16*::*mCherry* strain. Scale bars, 5μm. (F) MUT-16 western blot to assess protein levels in full-length and *mut-16* deletion strains. Expected sizes for MUT-16::mCherry::2xHA are 148 kD (full-length), 139 kD (ΔA), 137 kD (ΔB), 134 kD (ΔC), 138 kD (ΔD), 137 kD (ΔE), 138 kD (ΔF), 141 kD (ΔG), 132 kD (ΔH-I), 135 kD (ΔJ), 141 kD (ΔK), 138 kD (ΔL), 105 kD (ΔE-I), and 85 kD (ΔE-K). Approximately 200 synchronous adult animals were loaded per lane and actin was used as a loading control.

Each of the *mut-16* deletions were generated in the *mut-16*::*mcherry*::*2xHA* background. To determine which of these regions is required to promote foci formation, we examined the localization of *mut-16* in each deletion background by live imaging (Fig 3A–3E). Most deletions, including ΔA, ΔB, ΔD, ΔE, ΔF, ΔG, and ΔH-I, formed foci with similar intensity to the control (full-length) strain (Fig 3A and 3B). The ΔC mutation still formed *Mutator* foci, but had an overall reduced fluorescence (Fig 3A and 3C). It is unclear whether the reduced fluorescence intensity observed in the ΔC mutation is due to reduced expression or stability of MUT-16 specifically in the germline, but the overall MUT-16 protein levels are similar to full-length MUT-16 (Fig 3F). In contrast, ΔJ, ΔK, and ΔL displayed severely disrupted foci, though the overall expression of cytoplasmic MUT-16 was not reduced (Fig 3A and 3D). We also examined the two large deletions, ΔE-I and ΔE-K, which remove most of the IDR. Surprisingly, ΔE-I, which removes 387 amino acids (~37% of the protein) and encompasses a substantial portion of the IDR, does not affect MUT-16 localization, indicating that a large portion of the IDR is dispensable for foci formation. In contrast, ΔE-K does disrupt MUT-16 localization, which is not unexpected given that it includes J and K regions, which individually disrupted foci formation. All deletions were expressed at similar or higher levels than the full-length strain (Fig 3F), indicting that the reduced level of foci in ΔJ, ΔK, ΔL, and ΔE-K, is not due to lower protein expression. Together, these data indicate that C-terminal region of MUT-16 (J, K, and L) contains a region essential for foci formation.

### *Mutator* proteins are each recruited by different regions of MUT-16

In previous work we examined the requirements for *Mutator* foci formation [14]. In brief, MUT-16 is required for localization of MUT-2, MUT-7, RDE-2, MUT-14, and MUT-15, all of which localize independently of one another except for MUT-7, which requires RDE-2 for localization (Fig 1C). RRF-1 and RDE-8 have both been previously shown to colocalize and co-immunoprecipitate with MUT-16 but whether they directly interact with MUT-16 or interact via other *Mutator* complex proteins was unclear [14–16]. We also suspected the Zc3h12a-like ribonucleases NYN-1 and NYN-2 to be *Mutator* complex proteins because we had identified them in an IP-mass spectrometry experiment with MUT-16 (S1 Table). Additionally, Tsai *et al*. (2015) had identified them in a IP-mass spectrometry experiment with RDE-8, as well as demonstrated that the *nyn-1; nyn-2* double mutant displayed a marked reduction in WAGO-class 22G-RNAs, a phenotype similar to that of mutations in other *Mutator* complex components [16]. To determine whether NYN-1 localizes to the *Mutator* foci and to define the requirements for RRF-1, RDE-8, and NYN-1 localization, we generated GFP::RRF-1, mCherry::RDE-8, and mCherry::NYN-1 using CRISPR. Each protein forms distinct foci in germ cells and colocalizes with MUT-16, indicating that NYN-1 is indeed a component of the *Mutator* foci (S2A Fig). Tagged RRF-1, RDE-8, and NYN-1 strains were then crossed to strong loss-of-function mutations in other known *Mutator* foci components, including *mut-16*, *mut-2*, *rde-2*, *mut-14 smut-1*, and *mut-15*. Of the genes tested, only *mut-16* was required for RRF-1 localization (S2B Fig). Localization of RDE-8 and NYN-1 to *Mutator* foci requires both *mut-16* and *mut-15*. To determine if NYN-1 or RDE-8 were required for each other’s localization we further crossed mCherry::NYN-1 to *rde-8* mutants, and mCherry::RDE-8 to *nyn-1; nyn-2* double mutants. mCherry::NYN-1 was able to localize independently of RDE-8, whereas NYN-1 and NYN-2 were required for RDE-8 localization (S2B Fig).

**Fig 4:**
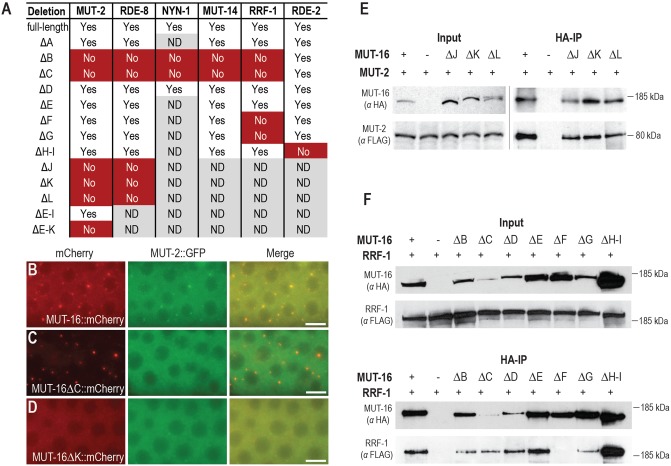
Distinct regions of MUT-16 recruit each of the other *Mutator* proteins. (A) Table indicates whether *mut-16* deletions disrupt MUT-2, RDE-8, NYN-1, MUT-14, RRF-1, and RDE-2 foci. Yes indicates foci present in the majority of animals, No indicates foci absent or severely disrupted, and ND indicates that strain was not constructed or scored. (B-D) MUT-16::mCherry and MUT-2::GFP expression and localization for control strain (B) or when ΔC (C) or ΔK (D) deletions have been introduced into the *mut-16*::*mCherry* strain. Scale bars, 5μm. (E) Immunoprecipitation and western blot of MUT-16::mCherry::2xHA (expected sizes between 135–141 kD for MUT-16 deletions and 148 kD for MUT-16 full length) and MUT-2::GFP::3xFLAG (83 kD). Left panels are total lysate from strains indicated above, and right panels are following HA immunoprecipitation. (F) Immunoprecipitation and western blot of MUT-16::mCherry::2xHA (expected sizes between 132–142 kD for MUT-16 deletions and 148 kD for MUT-16 full length) and GFP::3xFLAG::RRF-1 (219 kD). Top two panels are total lysate from strains indicated above, and bottom two panels are following HA immunoprecipitation. The equivalent of ~0.5% of starting material for the input fractions and ~20% of starting material for the IP fractions were loaded onto the gels.

To identify regions of the MUT-16 protein that are required for recruitment of other *Mutator* complex proteins we introduced the panel of *mut-16* deletions into the MUT-2::GFP, mCherry::RDE-8, mCherry::NYN-1, GFP::RRF-1, RDE-2::GFP, and MUT-14::GFP strains. Each of these six lines had a wild-type RNAi response in the presence of full-length *mut-16* (S3A Fig) [14]. All deletion strains were generated independently of one another and deletions of the same region in different strain backgrounds behaved similarly to one another with respect to their effect on MUT-16 localization and ability to respond to germline and somatic RNAi (S3B Fig). MUT-2::GFP foci were disrupted in the ΔB, ΔC, ΔJ, ΔK, and ΔL deletions (Fig 4A–4D and S4 Fig), however MUT-16 localization was also disrupted in ΔJ, ΔK, and ΔL (Figs 3A, 3D and 4D). To determine whether MUT-2 and MUT-16 could still interact at the molecular level in each deletion strain, we performed co-immunoprecipitation in each of the MUT-16 deletion backgrounds. The interaction between MUT-2 and MUT-16 was only disrupted in the ΔB and ΔC deletions, however, disruption of interactions between MUT-16 ΔC and other *Mutator* complex proteins could be at least partially due to the reduced expression of MUT-16 ΔC in the germline (S5 Fig). Despite the reduction in visible *Mutator* foci in ΔJ, ΔK, and ΔL, MUT-2 still physically interacts with MUT-16 at levels similar to the full-length control when these deletions are present (Fig 4E), indicating that ΔJ, ΔK, and ΔL regions are not required for recruitment of MUT-2 to the *Mutator* complex. This result suggests that when visible *Mutator* foci are disrupted in the ΔJ, ΔK, and ΔL mutants, many of the *Mutator* complex proteins may still interact in diffuse cytoplasmic complexes. We also observed that the MUT-16 protein is highly prone to degradation during the immunoprecipitation procedure. While full-length MUT-16 could be observed in most lanes, we also could detect multiple degradation products, with a prominent product of ~70kD in most lanes (S5 Fig). We did not observe substantial degradation with other proteins we worked with and suspect the highly disordered nature of MUT-16 may leave it more exposed to proteases during the immunoprecipitation procedure.

Similar to MUT-2 interaction, RDE-8, NYN-1, and MUT-14 depend on the ΔB and ΔC regions of MUT-16 for localization to the *Mutator* foci (Fig 4A and S4 Fig). In contrast, RDE-2 fails to localize to *Mutator* foci in the ΔH-I deletion, and RRF-1 is at least partially disrupted in ΔB, ΔC, ΔF, and ΔG mutants (Fig 4A and S4 Fig). Because RRF-1 foci are more difficult to detect than the other *Mutator* complex proteins, we also tested the physical interaction between MUT-16 and RRF-1 by co-immunoprecipitation in each of the MUT-16 deletion backgrounds. Only the ΔF mutant completely disrupted the interaction between MUT-16 and RRF-1, though the amount of RRF-1 immunoprecipitated by the ΔB, ΔC, and ΔG mutants was modestly reduced relative to full-length MUT-16 (Fig 4F). All together, these results suggest that different regions of MUT-16 are important for recruiting each of the *Mutator* complex proteins. These regions are separate and distinct from the C-terminal J, K, and L regions, which are important for *Mutator* foci formation.

### The C-terminal region of MUT-16 is sufficient for foci formation

**Fig 5:**
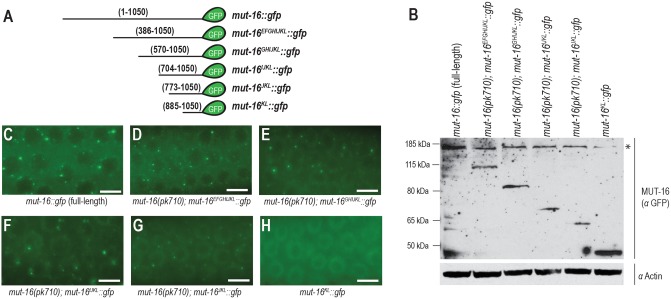
The C-terminal region of MUT-16 is sufficient for foci formation. (A) Diagram and amino acid coordinates of C-terminal regions of MUT-16 fused to GFP. (B) Western blot of full-length and C-terminal fragments of MUT-16::GFP probed with anti-GFP and anti-actin. A non-specific band (marked by asterisk) runs at a similar size to the full-length MUT-16::GFP. (C) Live imaging of full-length MUT-16::GFP, which forms foci throughout the germline. (D-G) C-terminal fragments of MUT-16::GFP form foci in the germline if they contain the minimal J, K, and L region (amino acids 773–1050) of the protein, but the number and size of foci increase with larger protein fragments. (H) The KL region (amino acids 885–1050) of MUT-16 fused to GFP is not sufficient for foci anywhere in the germline. All images are from the transition zone (leptotene/zygotene) region of the germline. Scale bars, 5μm.

The J, K, and L regions of MUT-16 are each necessary for robust MUT-16 foci formation; to determine whether they are also sufficient for foci formation, we generated a series of C-terminal fragments of MUT-16 fused to GFP and inserted them into the genome using MosSCI (Fig 5A and 5B) [33]. Full-length MUT-16 forms foci throughout the germline with the brightest foci present in the mitotic region and the leptotene/zygotene regions of the germline (Fig 5C) [14]. To guarantee that any visible foci are not seeded by full-length, untagged MUT-16, C-terminal MUT-16 fragments were introduced into a strain containing an early stop codon in the *mut-16* gene prior to imaging. All C-terminal GFP fusion constructs containing the JKL region (*mut-16*^*EFGHIJKL*^::*gfp*, *mut-16*^*GHIJKL*^::*gfp*, *mut-16*^*IJKL*^::*gfp*, and *mut-16*^*JKL*^::*gfp*) had visible MUT-16 foci, but as the fusion proteins became smaller (*mut-16*^*IJKL*^::*gfp* and *mut-16*^*JKL*^::*gfp*), the foci became less intense and fewer in number (Fig 5D–5G). *mut-16*^*KL*^::*gfp* did not form foci, even in the presence of an endogenous, full-length copy of MUT-16 and despite robust expression of this MUT-16 fragment (Fig 5B and 5H). These data are consistent with the JKL regions being necessary and sufficient for foci formation, but also indicate that additional regions of MUT-16 may help promote robust *Mutator* foci.

### MUT-16 foci formation is concentration dependent

**Fig 6:**
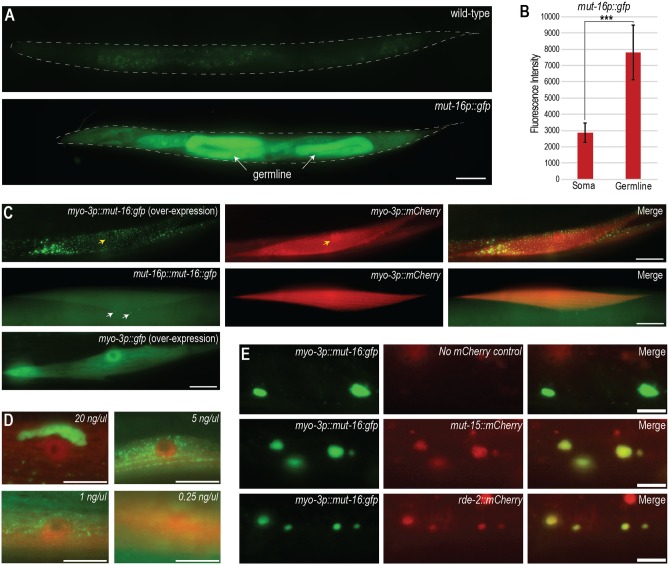
Concentration dependence of *Mutator* foci. (A) GFP expression in negative control (wild-type) and *mut-16p*::*gfp* L4 stage animals. The same exposure time was used for both images. Scale bar, 50μm. (B) Quantification of GFP fluorescence in the soma and the germline of *mut-16p*::*gfp* animals. Fluorescence intensity was calculated using ImageJ by tracing the germline and soma of 31 L4 stage animals and measuring the mean gray value. P values were calculated using a paired t-test (*** indicates p<0.001). (C) Top row: MUT-16::GFP overexpressed from the *myo-3* promoter forms foci in muscle cells marked with muscle-specific mCherry. Yellow arrow indicates the location of the nucleus. Middle row: Endogenously expressed MUT-16::GFP in muscle cells marked with muscle-specific mCherry does not form foci. White arrows point to MUT-16 foci in the neighboring germ cells. Bottom row: Overexpression of the GFP protein alone in muscle cells does not form foci. Scale bars, 10μm. (D) MUT-16::GFP in muscle cells labeled with *myo-3*::*mCherry*, where *myo-3p*::*mut-16*::*gfp* was injected at the indicated concentrations. Microscope exposure settings were changed as follows to account for variation in brightness between lines—top left: 0.02 sec exposure with neutral density filter at 10% transmittance; top right: 0.2 sec exposure; bottom left: 0.2 sec exposure; bottom right: 0.4 sec exposure. Scale bars, 10μm. (E) Top row: MUT-16::GFP overexpressed from the *myo-3* promoter forms foci. No mCherry proteins are expressed in this strain. Middle and bottom rows: Overexpression of MUT-16::GFP in muscle cells recruits MUT-15::mCherry (middle row) and RDE-2::mCherry (bottom row). Scale bars, 5μm.

We previously observed that *Mutator* foci form in the germline but not in somatic cells [15]. In the process of constructing an N-terminally GFP-3xFLAG-tagged MUT-16, a strain construction intermediate was a *mut-16* transcriptional reporter (*mut-16p*::*gfp*) at the endogenous *mut-16* locus [34]. While imaging *mut-16p*::*gfp*, we observed that, while GFP fluorescence is present throughout the animal, the germline is distinctly brighter, suggesting that MUT-16 may be expressed at higher levels in the germline compared to the somatic tissues (Fig 6A and 6B). Numerous studies have shown that phase separation is a concentration-dependent process [21–24,35]. We hypothesized that MUT-16 foci may form in germ cells but not in somatic cells because MUT-16 germ cell protein levels are above the concentration threshold at which MUT-16 phase separates to form foci. To test this hypothesis, we generated *myo-3p*::*mut-16*::*gfp*, which drives MUT-16 from a muscle-specific promoter and is expressed from a high-copy extrachromosomal array. This transgenic strain, in which the MUT-16 protein is overexpressed in muscle cells, has muscle-specific MUT-16::GFP foci (Fig 6C, top row). The foci vary in size, some being substantially larger than endogenously expressed MUT-16 foci. The co-expressed mCherry protein (*myo-3*::*mCherry*), which we also used as a marker for muscle cells, does not form foci but rather is expressed diffusely in both the cytoplasm and nucleus. Similarly, the GFP protein alone expressed under the *myo-3* promoter (*myo-3*::*gfp*) and injected at the same molar concentration as *myo-3p*::*mut-16*::*gfp*, is expressed diffusely in the cytoplasm and nucleus of muscle cells (Fig 6C, bottom row). A control strain which expressed only *myo-3p*::*mCherry* in the presence of *mut-16*::*gfp* expressed at the endogenous locus under its endogenous promoter (*mut-16p*::*mut-16*::*gfp*) did not have foci in muscle cells (Fig 6C, middle row). Rather, diffuse cytoplasmic expression of endogenous *mut-16p*::*mut-16*::*gfp* is visible in the muscle and throughout the animal, indicating that over-expression of the mCherry protein in the muscle does not drive MUT-16 into ectopic foci. To determine if the size or quantity of ectopic MUT-16 foci change at different protein concentrations, we injected the *myo-3p*::*mut-16*::*gfp* plasmid at 20 ng/ul, 5 ng/ul, 1 ng/ul or 0.25 ng/ul. While there was variability between independent lines isolated from each set of injections, generally lines from injections at the highest concentration (20 ng/ul) had larger and brighter foci than lines from lower concentrations (5 ng/ul, 1 ng/ul, or 0.25 ng/ul), whereas some lines from the lower concentration injections had no visible foci at all (Fig 6D). In some very high expressing 20 ng/ul lines, we observed large condensates of MUT-16::GFP protein that are no longer spherical (Fig 6D, top left). The presence of these larger structures could indicate that at very high concentrations MUT-16 is behaving more solid or gel-like, which has been seen previously for some IDR-containing proteins [24]. These data indicate that overexpression of MUT-16 in muscle cells is sufficient for somatic foci formation and suggest that MUT-16 can form foci in a concentration-dependent manner.

To determine whether the ectopic foci formed by overexpression of MUT-16 in the muscle can recruit other *Mutator* proteins, we generated extrachromosomal arrays overexpressing *myo-3p*::*mut-16*::*gfp* in strains carrying MosSCI lines expressing either *mut-15*::*mCherry* or *rde-2*::*mCherry*. In a control strain expressing only *myo-3p*::*mut-16*::*gfp*; there is very little mCherry signal overlapping the ectopic MUT-16 foci (Fig 6E, top row). In contrast, in the strains expressing *mut-15*::*mCherry* or *rde-2*::*mCherry* at endogenous levels, ectopic MUT-16 foci in the muscle recruit detectable levels of *mut-15*::*mCherry* or *rde-2*::*mCherry* (Fig 6E, middle and bottom rows). Thus overexpression of MUT-16 is sufficient to drive not just MUT-16, but also other *Mutator* complex proteins, into ectopic *Mutator* foci.

### Formation of *Mutator* foci depend on temperature and hydrophobic interactions

**Fig 7:**
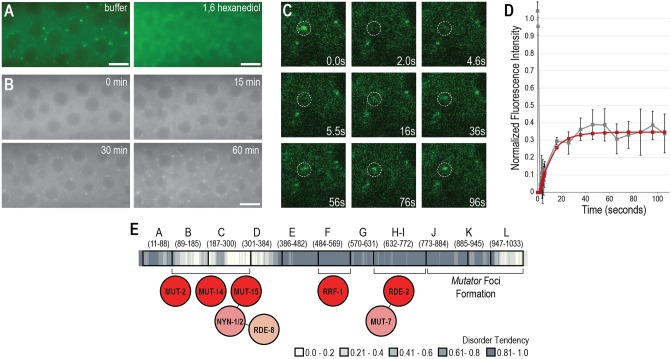
*Mutator* foci have liquid-like properties. (A) MUT-16::GFP are present in buffer control, but disappear in 5% 1,6-hexanediol. Scale bar, 5μm. (B) Images were collected at the indicated time points after MUT-16::GFP animals were heat-shocked for 6 hours at 30°C and then returned to room temperature (~21°C). MUT-16 foci are absent at the first time point (0 min), but begin to reform after ~15 min and look similar to untreated MUT-16 foci by 30–60 min. Scale bar, 5μm. (C) Time-lapse images show fluorescence recovery of MUT-16::GFP after a single *Mutator* focus is photobleached at 2.0 sec. (D) FRAP recovery curves for MUT-16::GFP. Raw data (gray line) is reported as a mean +/- SD (n = 5 granules). Data was fitted to a single exponential recovery curve (red line). (E) Schematic of MUT-16, indicating regions required for interaction with other *Mutator* complex proteins and for *Mutator* foci formation. Shading along the length of the MUT-16 protein represents disorder tendency calculated using IUPred (see Fig 1A).

Multiple studies have demonstrated that liquid-like condensates, but not solid aggregates, can be disrupted by aliphatic alcohols, such as 1,6-hexanediol [36–39]. These compounds disrupt weak, hydrophobic interactions, and have previously been shown to alter the permeability of the nuclear pore [40]. Addition of 5% 1,6-hexanediol to *C*. *elegans* gonads resulted in severely disrupted MUT-16 foci (Fig 7A). Because a liquid-like state requires weak molecular interactions whereas tighter interactions promote a solid state [41], these results support the hypothesis that *Mutator* foci have liquid-like properties.

Phase-separated condensates can also be perturbed by changes in temperature. For example, the DEAD-box helicase Ddx4, which is a component of germ granules, contains disordered regions that condense upon exposure to low temperatures and dissolve when returned to high temperatures [21]. Other disordered proteins behave in the opposite manner, condensing at high temperatures and dissolving at low temperatures [42]. To determine whether MUT-16 foci are temperature-sensitive, we subjected MUT-16::GFP animals to heat stress by placing them at 30°C for 6 hours. We returned the animals to room temperature (~21°C), and strikingly, the MUT-16 foci were no longer visible in the germ cells (Fig 7B and S6 Fig). However, within 15 minutes, some animals have already reformed MUT-16 foci (Fig 7B and S6 Fig). Over the full time course of 60 minutes at room temperature, many heat-shocked animals displayed no discernable differences in foci presence and intensity from animals raised at permissive temperatures (Fig 7B, S6 Fig, and S1 Movie). These data indicate that MUT-16 foci can change in response to environmental conditions, such as temperature.

### *Mutator* foci recover after photobleaching

Germ granule proteins, such as PGL-1, PGL-3, and LAF-1 in *C*. *elegans* P granules, recover rapidly after photobleaching indicating that the internal components of the granule can rearrange, similar to molecules in a liquid state [22,28,43,44]. To determine if MUT-16 foci are similarly liquid-like, we performed fluorescence recovery after photobleaching (FRAP) experiments. Due to the small size of *Mutator* foci (<500 nm), we chose to bleach entire foci and measure the recovery of MUT-16 to the foci from the surrounding cytoplasm. MUT-16::GFP foci recovered rapidly after photobleaching, *t*_1/2_ = 7.2 ± 1.0 seconds (SEM, n = 5). Recovery only reaches ~35% of pre-bleached intensity (Fig 7C and 7D and S1 Movie), indicating that there may be both a mobile fraction of MUT-16 that can exchange quickly between *Mutator* foci and the cytoplasm, and an immobile fraction that exchanges very slowly or not at all. Incomplete recovery of fluorescence has previously been observed for *in vivo* FRAP of P granules and of the adjacent and recently discovered Z granules [43–45]. Fluorescence recovery data, together with the dependence of *Mutator* foci on concentration, temperature, and hydrophobic interactions, suggest that *Mutator* foci have properties of a phase-separated condensate.

## Discussion

### Functional subdivision of MUT-16

MUT-16 function can be subdivided across different regions of the protein (Fig 7E). The largest structured region (specifically the B-C region) of MUT-16 recruits multiple proteins, including the nucleotidyl transferase MUT-2, the DEAD-box RNA helicase MUT-14, and the protein of unknown function MUT-15, whose localization requirements we can infer based on the localization of the Zc3h12a-like ribonucleases RDE-8 and NYN-1 (Fig 4A and 4C, and S2B Fig). MUT-2, MUT-14, and MUT-15 likely interact directly with MUT-16, as we can robustly detect these interactions by immunoprecipitation and no other unknown proteins have been identified as part of this complex by IP-mass spectrometry experiments (Fig 4, S5 Fig and S1 Table) [14]. Since MUT-15 recruits NYN-1, NYN-2 and RDE-8, at least six *Mutator* complex proteins localize to *Mutator* foci through the B-C region of MUT-16 (S2B Fig and Fig 7E). It remains to be determined whether a single MUT-16 protein can interact with all of these proteins concurrently.

Interestingly, while we initially hypothesized that the IDR would be important for promoting *Mutator* foci formation, we found regions of disorder also function to recruit proteins to the *Mutator* complex. In particular, region F recruits the RdRP protein RRF-1 and the H-I region recruits the exonuclease MUT-7 through its interaction with RDE-2. The regions that directly recruit *Mutator* complex proteins correlate with regions required for robust RNAi in somatic tissues (Fig 2). Specifically, *mut-16*ΔC, ΔF, and ΔH-I have somatic RNAi defects nearly as severe as a *mut-16* null allele. *mut-16*ΔL also has similarly severe somatic RNAi, however unlike ΔC, ΔF, and ΔH-I, deletion of the L region does not disrupt the interaction of MUT-16 with any other *Mutator* complex proteins of which we are aware. Since we cannot detect *Mutator* foci in the soma, we do not yet know how the loss of the L region affects *Mutator* complex formation or function in somatic cells. In addition, it is unclear why the remaining regions of MUT-16 are required for germline but not somatic RNAi. It is possible that they recruit yet uncharacterized germline-specific RNAi factors, or may facilitate currently unexamined interactions between primary and secondary Argonaute proteins and the *Mutator* complex. Overall, this data suggests that at least some of the most severe defects in somatic RNAi that occur after deleting portions of the *mut-16* gene stem from loss of recruitment of specific proteins to the *Mutator* complex.

Our fusion of C-terminal fragments of MUT-16 to GFP, demonstrates that a minimal region comprised of amino acids 773–1050 (JKL region) is sufficient for *Mutator* foci formation (Fig 5). This region is ~70% disordered, but contains the structured L region that is also key to the formation of *Mutator* foci. We did observe that including additional regions of the MUT-16 protein, specifically a larger portion of the disordered region, can promote increased size and number of *Mutator* foci. Interestingly, the regions with the highest glutamine and proline content are centered on the H-I and J regions of MUT-16, only partially overlapping this region critical for *Mutator* foci formation. This finding suggests that isolated stretches of disordered amino acids are not sufficient to mediate phase separation, but require the neighboring protein environment to promote *Mutator* foci formation.

### MUT-16 foci have properties of a phase-separated condensate

Only recently has it become clear that many biological processes involve intracellular phase transitions, from spindle assembly to heterochromatin formation [37,42,46]. Proteins that are heterogeneous in conformation, such as proteins containing IDR, typically drive phase separation by providing a flexible platform for non-covalent interactions with nearby disordered proteins and/or ribonucleic acids. Other conditions such as protein or RNA concentration, post-translational modification, or changes in environmental conditions such as salt concentration or temperature can modulate these transitions [47]. Interestingly, we have been unable to co-immunoprecipitate MUT-16 with itself, which, while not conclusive, suggests that MUT-16 does not form strong intermolecular interactions and rather promotes *Mutator* foci formation via many weak or transient interactions.

Based on our studies of MUT-16, we propose that *Mutator* foci are phase-separated membrane-less organelles with liquid-like properties rather than a solid or aggregated structure, with five lines of evidence supporting this hypothesis. First, *Mutator* foci are roughly spherical in shape, as would be expected of a liquid compartment due to the cohesive forces of the surface layer (i.e. surface tension). Second, *Mutator* foci dissolve in the aliphatic alcohol, 1,6-hexanediol, which disrupts only weak, hydrophobic interactions but not solids. Third, MUT-16::GFP foci recover rapidly, though incompletely, after photobleaching, indicating that a mobile fraction of MUT-16 can exchange freely between the *Mutator* foci and the cytoplasm. Fourth, *Mutator* foci only assemble when concentrations rise above a certain threshold, which is a hallmark of phase-separated condensates. And fifth, *Mutator* foci formation can be modulated by changes in temperature—increased entropy at higher temperatures can counteract phase separation and promote mixing of the condensed and bulk phases. The latter two points indicate that *Mutator* foci may be sensitive to intracellular and extracellular conditions, which could allow foci formation to be fine-tuned based on cell type or environmental conditions.

A liquid-like nature of *Mutator* foci could allow for some proteins and RNA to freely exchange in and out of the compartment, while still maintaining a high concentration of factors required for small RNA amplification. Furthermore, the close juxtaposition of *Mutator* foci with nuclear pores and P granules, which also have liquid-like properties, suggests a model of RNA silencing where adjacent membrane-less organelles can exchange proteins and RNAs, but have intrinsic properties that make them immiscible with one another. A similarly close juxtaposition has been observed between P bodies and stress granules, and these types of interactions may reflect differences in surface tension of the two types of condensates [48,49]. Further characterization both *in vivo* and *in vitro* will be necessary to fully elucidate how *Mutator* foci form and interact with neighboring membrane-less organelles.

### Properties of the *Mutator* complex in the soma vs. germline

Why are *Mutator* foci present in the germline but not the soma? The answer may lie in the unique biophysical properties of MUT-16, as well as in the concentration of and local environment surrounding MUT-16 in the germline. By concentrating *Mutator* complex proteins to the cytoplasmic side of nuclear pores, *Mutator* foci may be poised to capture deleterious RNAs during nascent RNA export. It is unclear why a similar mechanism is not also in place in somatic cells, but perhaps such a robust RNA silencing mechanism is not necessary in the soma as these cells will not be passed to the next generation.

*Mutator* foci in the germline are exclusively associated with the nuclear periphery, until the germ cells become oocytes and the P granules detach from the nuclei and become cytoplasmic. *Mutator* foci and some nuclear pore components move with the P granules into the cytoplasm [14,43,50]. We do not yet know whether *Mutator* foci can interact directly with nuclear pores or if this interaction is mediated by the P granule or another unknown factor, however knockdown of P granule components does not disrupt the perinuclear localization of *Mutator* foci [14]. Interestingly, while some of the MUT-16 foci in *myo-3p*::*mut-16*::*gfp* are adjacent to the nuclear periphery, most are not (Fig 6C). It is unclear whether the lack of perinuclear association in the muscle cells is due to the lack of a specific anchoring factor present only in germ cells, or perhaps the anchoring factor (for example, nuclear pores) is present but limited in supply relative to the substantial overexpression of MUT-16.

In conclusion, our results demonstrate that MUT-16 serves as a scaffold for assembly of *Mutator* foci and suggest that these foci form through liquid-liquid phase separation. The association of *Mutator* foci with P granules and nuclear pores suggests a model whereby newly transcribed mRNAs pass through nuclear pores into the P granule, where mRNAs are marked for silencing and targeted to the neighboring *Mutator* foci. Subsequently, siRNAs synthesized in the *Mutator* foci and perhaps secondary Argonaute proteins can transit back into the P granules to target additional complementary mRNAs for silencing. Thus, we propose that RNA silencing in germ cells may depend on RNA transit through phase-separated liquid compartments that concentrate RNA silencing enzymes.

## Methods

### Strains

The *C*. *elegans* wild-type strain is N2. Worms were cultured at 20°C according to standard conditions unless otherwise stated [51]. Strains used include WM30 (*mut-2(ne298)* I), NL3531(*rde-2(pk1657)* I), NL1810 (*mut-16(pk710)* I), FX04844 (*nyn-2(tm4844)* I), NL1820 (*mut-7(pk720)* III), HT1593 (*unc-119(ed3)* III), EG4322 (*ttTi5605* II; *unc-119(ed9)* III), GE24 *(pha-1(e2123)* III), FX05004 (*nyn-1(tm5004)* IV), FX02252 (*rde-8 (tm2252)* IV), GR1948 (*mut-14(mg464) smut-1(tm1301)* V), and GR1747(*mut-15(tm1358)* V). All mutants were outcrossed at least 4x prior to any analysis. New strains made for this project are listed in S2 Table.

### Plasmid and strain construction

All GFP and mCherry constructs were designed for insertion at the endogenous loci by CRISPR genome editing except the C-terminal fragments of *mut-16* fused to GFP (Fig 5), which were integrated by Mos-mediated single-copy transgene insertion (MosSCI) [33]. For all CRISPR insertions of GFP or mCherry, we generated homologous repair templates using the primers listed in S3 Table. To create the *mut-16*::*gfp*::*3xFLAG* repair template, we first generated a GFP with C-terminal 3xFLAG tag and internal Floxed *Cbr-unc-119(+)*, and inserted into the Kanamycin-resistant backbone of pDONR221 by isothermal assembly [52]. This cassette was then flanked with ~1.5-2kb of sequence from either side of the *mut-16* stop codon, again by isothermal assembly. A similar design was used to create all mCherry repair templates. We first generated a *2xHA*::*mCherry[w/internal Floxed Cbr-unc-119(+)]*::*2xHA* construct by cloning *mCherry[w/internal Floxed Cbr-unc-119(+)]* into XhoI/SpeI-digested pBluescript SK(-). The *mCherry[w/internal Floxed Cbr-unc-119(+)]* was a gift from the lab of Jeremy Nance (NYU). BamHI and BglII sites were engineered into the mCherry construct just following the ATG or just preceding the stop codon, respectively. This construct was sequentially digested with BglII, then BamHI; each digest was followed by ligation of a 2xHA oligo. This construct was then used as a PCR template to generate *mCherry*::*2xHA[w/internal Floxed Cbr-unc-119(+)]* or *2xHA*::*mCherry[w/internal Floxed Cbr-unc-119(+)]*, and assembled with ~1kb of sequence from either side of the *mut-16* stop codon, or the *rde-8* and *nyn-1* start codons, respectively. *mut-2*::*gfp*::*3xFLAG*, *rde-2*::*gfp*::*3xFLAG*, *gfp*::*3xFLAG*::*rrf-1*, and *gfp*::*3xFLAG*::*mut-16* (for *mut-16p*::*gfp*) repair templates were assembled into pDD282 (GFP/3xFLAG with self-excising cassette, Addgene #66823) according to published protocols [34]. To protect the repair template from cleavage, we introduced silent mutations at the site of guide RNA targeting by incorporating these mutations into one of the homology arm primers or, if necessary, by performing site-directed mutagenesis [53].

The *mut-16* guide RNA was cloned into PU6::*unc-119*_sgRNA (Addgene #46169) by site-directed mutagenesis [54]. All other guide RNA plasmids were generated by ligating oligos containing the guide RNA sequence into BsaI-digested pRB1017 (Addgene #59936) [55]. Guide RNA sequences are provided in S3 Table.

C-terminal fragments of *mut-16* fused to GFP were cloned into targeting vectors for MosSCI. The endogenous promoter and 3’UTR were amplified, along with either full-length coding sequence or C-terminal fragments of the coding sequence (S3 Table). These amplicons were inserted along with GFP by isothermal assembly into SpeI-digested pCFJ151 (Addgene #19330) [33,52]. A similar strategy was used to generate *myo-3p*::*mut-16*::*gfp*, except with the *mut-16* promoter replaced by the *myo-3* promoter.

CRISPR injections were performed according to published protocols [34,53,56]. GFP/mCherry CRISPR injection mixes included 10–25 ng/μl repair template, 50 ng/μl guide RNA, 50 ng/μl *eft-3p*::*cas9-SV40_NLS*::*tbb-2 3'UTR* (Addgene # 46168), 2.5–10 ng/μl GFP or mCherry co-injection markers, and 10 ng/μl *hsp-16*.*1*::*peel-1* negative selection (pMA122, Addgene #34873). *mut-16*::*gfp*::*3xFLAG* and all mCherry constructs were injected into HT1593 (*unc-119(ed3)* III). Floxed *Cbr*-*unc-119(+)* cassettes were later excised using *eft-3p*::*Cre* (pDD104, Addgene #47551) [53]. *mut-2*::*gfp*::*3xFLAG*, *rde-2*::*gfp*::*3xFLAG*, and *gfp*::*3xFLAG*::*rrf-1* were injected into the wild-type strain. *mut-16* deletion injection mixes included 50 ng/μl oligo repair template, 25 ng/μl each of two *mut-16* guide RNAs, 50 ng/μl pha-1 repair oligo and 50 ng/μl *eft-3p*:*Cas9 + pha-1 guide RNA* (pJW1285, Addgene #61252). Mixtures were injected into *pha-1(e2123)* mutant animals already carrying *mut-16*::*gfp*::*3xFLAG* or *mut-16*::*mCherry*::*2xHA* and other fluorescently-tagged *Mutator* complex proteins and *mut-16* deletions were identified by PCR according to *pha-1* co-conversion protocol [56].

For MosSCI injections, we integrated transgenes into the *ttTi5605 MosI* site in strain EG4322 (Ch. II) following a published MosSCI protocol [33]. Injection mixes contained 50 ng/μl MosSCI-targeting vector, 50 ng/μl *eft-3p*::*Mos1 transposase* (pCFJ601, Addgene #34874), 10 ng/μl *rab-3p*::*mCherry* (pGH8, Addgene #19359), 2.5 ng/μl *myo-2p*::*mCherry* (pCFJ90, Addgene #19327), 5 ng/μl *myo-3p*::*mCherry* (pCFJ104, Addgene #19328), and 10 ng/μl *hsp-16*.*1*::*peel-1* negative selection (pMA122, Addgene #34873). Extra-chromosomal arrays were generated as follows: 20ng/ul *myo-3p*::*mut-16*::*gfp*, 5 ng/μl *myo-3p*::*mCherry* (pCFJ104) and 70 ng/ul pBluescript injected into HT1593—*unc-119(ed3)* for *cmpEx76 and cmpEx89*; 5 ng/ul *myo-3p*::*mut-16*::*gfp*, 5 ng/μl *myo-3p*::*mCherry* (pCFJ104) and 70 ng/ul pBluescript injected into HT1593—*unc-119(ed3)* for *cmpEx90*; 1 ng/ul *myo-3p*::*mut-16*::*gfp*, 5 ng/μl *myo-3p*::*mCherry* (pCFJ104) and 70 ng/ul pBluescript injected into HT1593—*unc-119(ed3)* for *cmpEx91; 0*.*2*5 ng/ul *myo-3p*::*mut-16*::*gfp*, 5 ng/μl *myo-3p*::*mCherry* (pCFJ104) and 70 ng/ul pBluescript injected into HT1593—*unc-119(ed3)* for *cmpEx92*; 5 ng/μl *myo-3p*::*mCherry* (pCFJ104) and 70 ng/ul pBluescript injected into the *mut-16(cmp3[mut-16*::*gfp*::*3xFLAG])* strain for *cmpEx79*; 7.5 ng/ul *myo-3p*::*gfp* (pPD118.20) and 70 ng/ul pBluescript into the wild-type strain for *cmpEx88*; and 20ng/ul *myo-3p*::*mut-16*::*gfp* and 70 ng/ul pBluescript injected into HT1593—*unc-119(ed3)*, *mut-15*::*mCherry*, and *rde-2*::*mCherry* strains for *cmpEx76*, *cmpEx86*, and *cmpEx87*.

### Antibody staining and imaging

*C*. *elegans* were imaged in M9 buffer with sodium azide to prevent movement. In some cases, to obtain higher quality images, animals were dissected prior to mounting. For scoring presence or absence of foci, deletions were blinded and scored by multiple individuals. A minimum of 10 animals were scored for each strain. Imaging was performed on a DeltaVision Elite microscope (GE Healthcare) using a 60x N.A. 1.42 oil-immersion objective. When data stacks were collected, three-dimensional images are presented as maximum intensity projections. Images were pseudocolored using the SoftWoRx package or Adobe Photoshop.

For quantification of somatic and germline fluorescence intensity in the *mut-16p*::*gfp* strain, 31 age-matched L4 animals were imaged using identical microscope settings. The somatic and germline tissue was traced in ImageJ and mean gray value was calculated for each region. 1,6-hexanediol treatment was performed by dissecting adult MUT-16::GFP animals in M9 buffer with or without the addition of 5% 1,6-hexanediol and imaged immediately following dissection. For heat stress experiment, plates of L4 stage MUT-16::GFP animals were wrapped in Parafilm and placed in a 30°C incubator for 6 hours. We then removed the plates from the incubator, immediately transferred the animals to slides, and began imaging. No more than 5 minutes elapsed between removal from 30°C and collection of the first time point (Fig 7B, 0 min). Images were captured every 3 minutes for 60 minutes at room temperature (~21°C).

Fluorescence recovery after photobleaching (FRAP) was performed on a Leica SP8 Falcon laser scanning confocal microscope using a 63x N.A. 1.4 oil-immersion objective. Two images were acquired prior to bleaching, followed by bleaching, then 10 images at 0.44 second intervals and 10 additional images at 10 second intervals. Data was analyzed using the Leica Application Suite X software.

### Immunoprecipitation and western blots

Immunoprecipitations were performed as previously described [14]. ~40,000 synchronized adult *C*. *elegans* (~68 h at 20°C after L1 arrest) were collected, frozen in liquid nitrogen, and homogenized using a mortar and pestle. After further dilution into lysis buffer (1:10 packed worms:buffer), insoluble particulate was removed by centrifugation and a sample was taken as “input.” The remaining lysate was used for the immunoprecipitation. HA-tagged proteins were immunoprecipitated using anti-HA affinity matrix (ThermoFisher 26181).

For Western blots, proteins were resolved on 4–12% Bis-Tris polyacrylamide gels (ThermoFisher), transferred to nitrocellulose membranes, and probed with anti-HA 1:1,000 (Roche 12013819001), anti-FLAG 1:1,000 [M2 clone] (Sigma-Aldrich F1804), anti-actin 1:10,000 (Abcam ab3280), or anti-GFP 1:100 (Riken BRC JFP-J5) [57]. Secondary HRP antibodies were purchased from ThermoFisher.

We observed that MUT-16 was often substantially degraded during the immunoprecipitation procedure. We attempted to remedy this problem by reducing the time samples spent in lysis buffer, shortening the immunoprecipitation step, and increase the amount of protease inhibitor. These steps resulted in little improvement of full-length MUT-16 yield in both input and IP samples. We also noted that the MUT-16 degradation product was present in full-length and all MUT-16 deletion mutants except for ΔH-I. Given that MUT-16 is tagged at the C-terminus, the deletions preceding ΔH-I all form degradation products of the same size, and the deletions following ΔH-I all vary in size in proportion to the relative deletion sizes, we suspect that the degradation site in MUT-16 is somewhere in the ΔH-I region (S5 Fig).

For mass spectrometry experiments, immunoprecipitation was performed as described above, starting with ~500,000 synchronized adult *C*. *elegans* (~68 h at 20°C after L1 arrest) for each sample. GFP and FLAG immunoprecipitation was performed using anti-GFP affinity matrix [RQ2 clone] (MBL International D153-8) and anti-FLAG affinity matrix [M2 clone] (Sigma-Aldrich A2220). After immunoprecipitation, samples were precipitated using the ProteoExtract Protein Precipitation Kit (EMD Millipore 539180) and submitted to the Taplin Mass Spectrometry facility at Harvard Medical School for protein identification.

### RNAi assays

For RNAi assays, L1 animals were fed *E*. *coli* expressing dsRNA against *pos-1*, *lin-29*, or *nhr-23* [58]. For *pos-1*, animals were scored 4 days later for hatching of the F1 embryos. For *lin-29* or *nhr-23*, animals were scored 2–3 d later for vulval bursting or larval arrest, respectively. To quantify intermediate phenotypes of *nhr-23* and *lin-29* RNAi, we established two subcategories to distinguish between partial RNAi defects (animals are smaller than wild-type with few or no eggs as adults, and for *lin-19*, often have a protruding vulva) and total RNAi defects (animals are phenotypically wild-type).

## Supporting information

S1 Fig Distribution of Q, N, and P residues in MUT-16 orthologs. The frequency of Q, N, and P residues were analyzed in *C*. *elegans* MUT-16 (WP:CE40347), *C*. *briggsae* MUT-16 (BP:CBP44329), *C*. *remanei* MUT-16 (RP:RP48608), and *C*. *japonica* MUT-16 (JA:JA63728) proteins. Residues were counted in amino acid 100-mers, starting at position one, shifting 10 residues at a time, and displayed as stacked columns. Indicated residue positions are the mid-point of the 100-mer.
(PDF)

S2 Fig Requirements for RRF-1, NYN-1, and RDE-8 localization. (A) GFP::RRF-1, mCherry::NYN-1, and mCherry::RDE-8 each colocalize with MUT-16. (B) GFP::RRF-1, mCherry::NYN-1, and mCherry::RDE-8 were introduced into each of the indicated mutant backgrounds. RRF-1 foci were disrupted in *mut-16* mutants, NYN-1 were disrupted in *mut-16* and *mut-15* mutants, and RDE-8 foci were disrupted in *mut-16*, *mut-15*, and *nyn-1; nyn-2* double mutants. All images are from the transition zone (leptotene/zygotene) region of the germline. Scale bars, 5μm.
(PDF)

S3 Fig Response of GFP- and mCherry-tagged strains to somatic and germline RNAi. (A) Deletion strain controls were assessed for RNAi response to somatic (*nhr-23* and *lin-29*) or germline (*pos-1*) RNAi. For somatic RNAi, P0 animals were categorized as having fully penetrant (red bars) or intermediate RNAi defects (gray bars). Intermediate effects were categorized as non-thriving, slow moving adults with eggs for *nhr-23* and as adults with protruding vulvas for *lin-29*. For *pos-1*, the F1 eggs and hatched larvae were counted to calculate % viable progeny from treated P0 animals. Weighted means and standard deviations were calculated from three independent RNAi trials of n = ~20 for *nhr-23* and *lin-29*, and n = ~140 F1 eggs from 4 P0 adults for *pos-1*. (B) Deletion strains were assessed for RNAi response and categorized as having either fully penetrant RNAi defects (Rde, red boxes), intermediate RNAi defects (Weak, pink boxes), or wild-type response (WT, white boxes). ND indicates that strain was not constructed or scored. For *nhr-23*, fully penetrant RNAi defects are strains where >65% of animals are non-arrested and healthy, intermediate RNAi defects are strains where >35% are non-arrested (either healthy or sick), and wild-type response are strains where ≤35% are non-arrested (either healthy or sick). For *lin-29*, fully penetrant RNAi defects are strains where >70% of animals were scored as non-burst, intermediate RNAi defects are strains where 35%-70% of animals were scored as non-burst, and wild-type response are strains where <35% are non-burst. For *pos*-1, fully penetrant RNAi defects are strains where >75% F1s are viable, intermediate RNAi defects are strains where 10–75% F1 are viable, and wild-type response are strains <10% F1 are viable.
(PDF)

S4 Fig Distinct MUT-16 regions are required for the localization of the other *Mutator* proteins. Live imaging of MUT-16 and other *Mutator* proteins in each of the *mut-16* deletion backgrounds. All images are from the transition zone (leptotene/zygotene) region of the germline. Scale bars, 5μm.
(PDF)

S5 Fig Regions B and C of MUT-16 are required for interaction with MUT-2. Immunoprecipitation and western blot of MUT-16::mCherry::2xHA (expected sizes between 132–141 kD for MUT-16 deletions and 148 kD for MUT-16 full length) and MUT-2::GFP::3xFLAG (83 kD). Top three panels are total lysate from strains indicated above, and bottom two panels are following HA immunoprecipitation. Note that in some cases, full-length MUT-16 was degraded beyond the detection limit of the western blot in the input sample, but was still present following immunoprecipitation. The equivalent of ~0.5% of starting material for the input fractions and ~20% of starting material for the IP fractions were loaded onto the gels.
(PDF)

S6 Fig MUT-16 foci are temperature dependent. Representative images from the early pachytene region after L4 animals were subjected to 30°C heat shock for 6 hours and allowed to recover on plates at room temperature (~21°C) for the indicated amount of time. All images are from different animals to demonstrate the variability in foci presence and intensity at each time point. Scale bars, 5μm.
(PDF)

S1 Movie *Mutator* foci are temperature dependent. Time-lapse imaging of MUT-16::GFP animals following 6 hours at 30°C and a return to room temperature (~21°C). Elapsed time at room temperature is indicated in minutes (bottom left).
(AVI)

S2 Movie *Mutator* foci recover rapidly after photobleaching. Time-lapse imaging of MUT-16::GFP fluorescence recovery after photobleaching. Elapsed time is indicated in seconds (bottom right). Photobleaching occurs at 2 seconds and dashed circle indicates bleached *Mutator* focus.
(AVI)

S1 Table Peptide counts and percent coverage for *Mutator* proteins identified by mass spectrometry following FLAG or GFP immunoprecipitation from wild-type and MUT-16::GFP::FLAG lysates. (XLSX)

S2 Table Strains generated for this study. (XLSX)

S3 Table Oligonucleotides sequences used in this study. (XLSX)
