# Supplementary material for: Distinct regions of the intrinsically disordered protein MUT-16 mediate assembly of a small RNA amplification complex and promote phase separation of Mutator foci
Source: PLoS Genet. 2018 Jul 23;14(7):e1007542. doi: 10.1371/journal.pgen.1007542 (PMC6072111; doi:10.1371/journal.pgen.1007542)
Supplement: S1 Fig — The frequency of Q, N, and P residues were analyzed in C. elegans MUT-16 (WP:CE40347), C. briggsae MUT-16 (BP:CBP44329), C. remanei MUT-16 (RP:RP48608), and C. japonica MUT-16 (JA:JA63728) proteins. Residues were counted in amino acid 100-mers, starting at position one, shifting 10 residues at a time, and displayed as stacked columns. Indicated residue positions are the mid-point of the 100-mer. (PDF) [file pgen.1007542.s001.pdf]

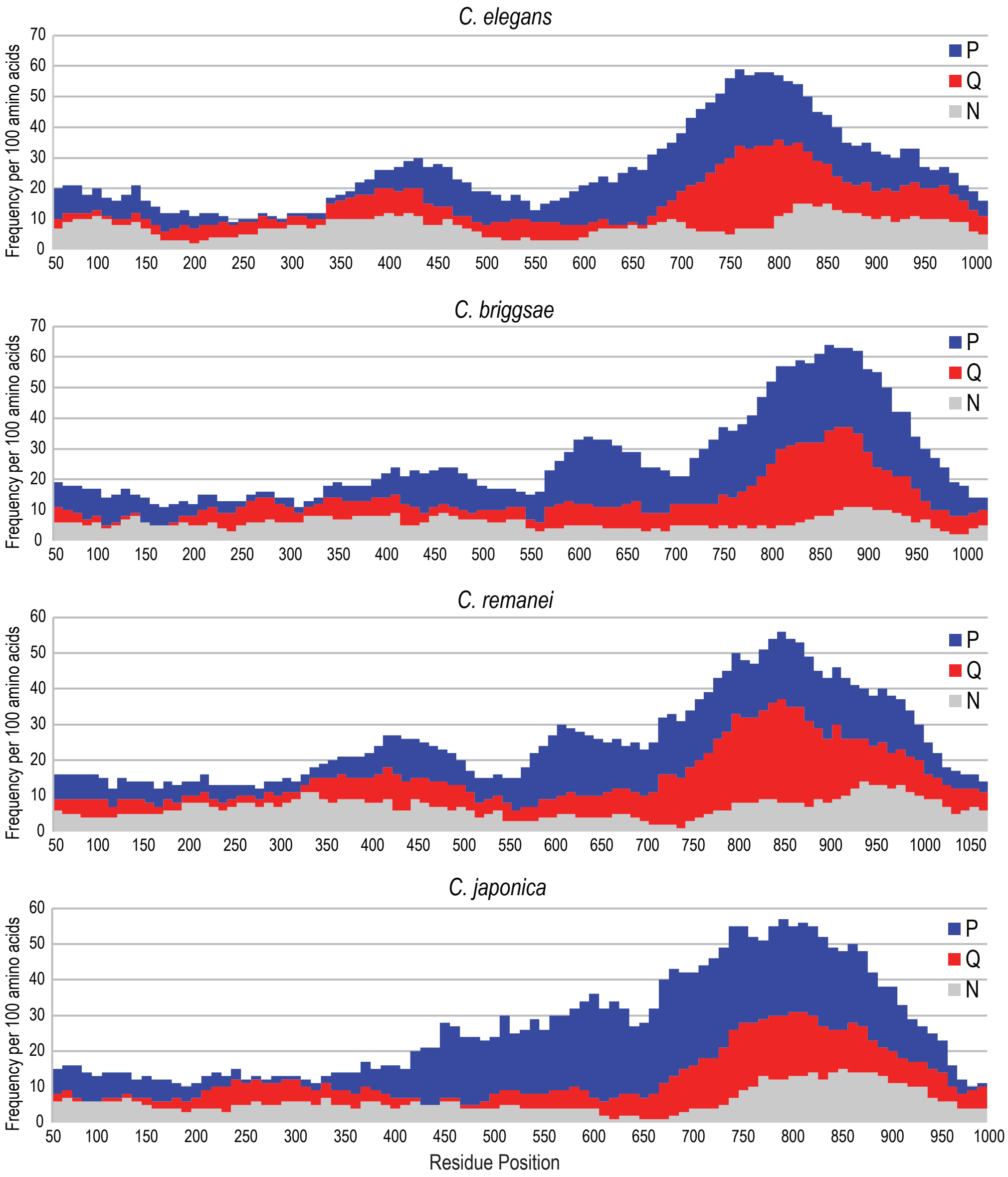

S1 Fig. Distribution of Q, N, and P residues in MUT-16 orthologs.
The frequency of Q, N, and P residues were analyzed in *C. elegans* MUT-16 (WP:CE40347), *C. briggsae* MUT-16 (BP:CBP44329), *C. remanei* MUT-16 (RP:RP48608), and *C. japonica* MUT-16 (JA:JA63728) proteins. Residues were counted in amino acid 100-mers, starting at position one, shifting 10 residues at a time, and displayed as stacked columns. Indicated residue positions are the mid-point of the 100-mer.
