# Supplementary material for: Distinct regions of the intrinsically disordered protein MUT-16 mediate assembly of a small RNA amplification complex and promote phase separation of Mutator foci
Source: PLoS Genet. 2018 Jul 23;14(7):e1007542. doi: 10.1371/journal.pgen.1007542 (PMC6072111; doi:10.1371/journal.pgen.1007542)
Supplement: S2 Fig — (A) GFP::RRF-1, mCherry::NYN-1, and mCherry::RDE-8 each colocalize with MUT-16. (B) GFP::RRF-1, mCherry::NYN-1, and mCherry::RDE-8 were introduced into each of the indicated mutant backgrounds. RRF-1 foci were disrupted in mut-16 mutants, NYN-1 were disrupted in mut-16 and mut-15 mutants, and RDE-8 foci were disrupted in mut-16, mut-15, and nyn-1; nyn-2 double mutants. All images are from the transition zone (leptotene/zygotene) region of the germline. Scale bars, 5μm. (PDF) [file pgen.1007542.s002.pdf]

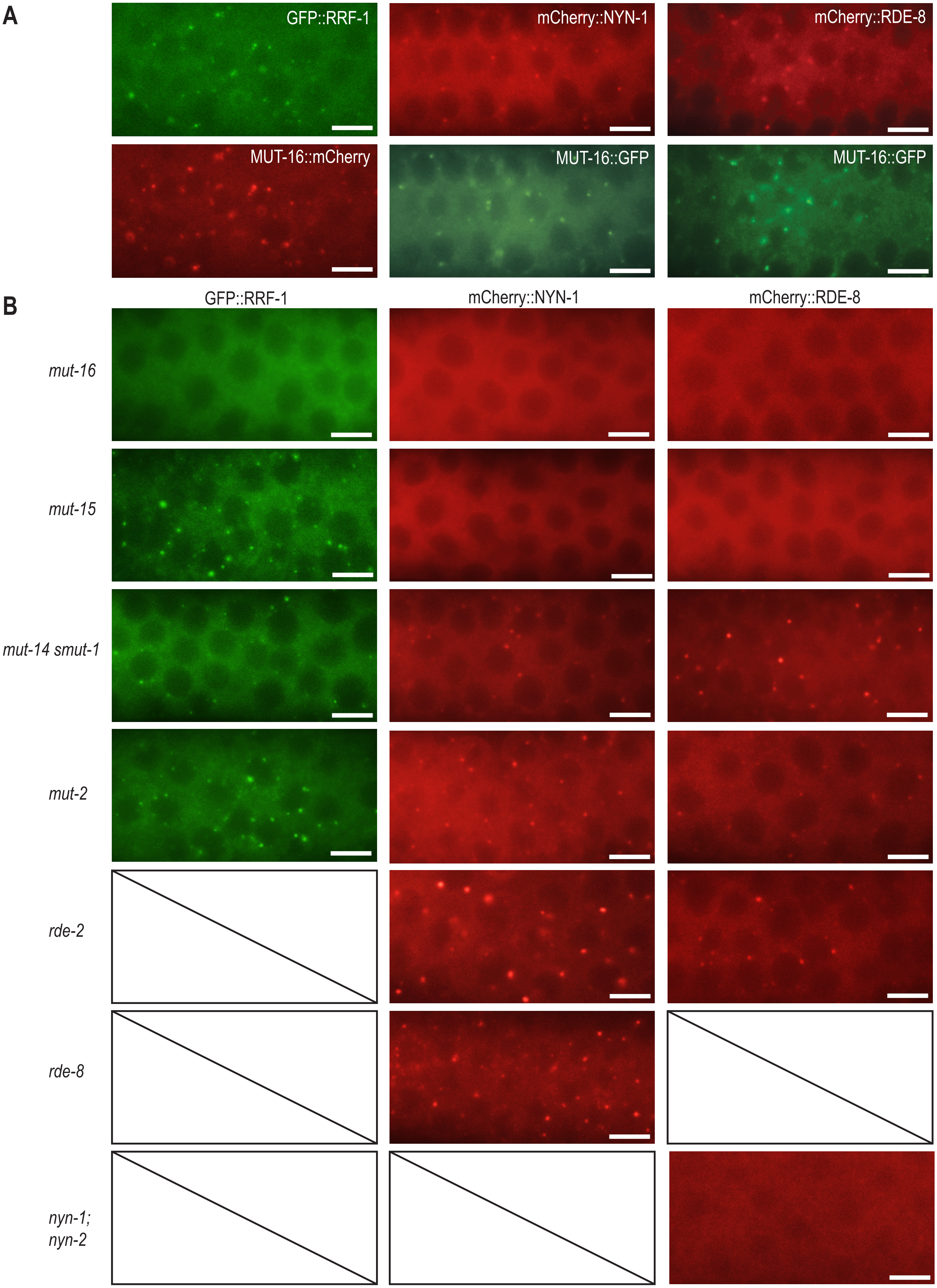

S2 Fig. Requirements for RRF-1, NYN-1, and RDE-8 localization.

(A) GFP::RRF-1, mCherry::NYN-1, and mCherry::RDE-8 each colocalize with MUT-16.

(B) GFP::RRF-1, mCherry::NYN-1, and mCherry::RDE-8 were introduced into each of the indicated mutant backgrounds. RRF-1 foci were disrupted in *mut-16* mutants, NYN-1 were disrupted in *mut-16* and *mut-15* mutants, and RDE-8 foci were disrupted in *mut-16*, *mut-15*, and *nyn-1; nyn-2* double mutants. All images are from the transition zone (leptotene/zygotene) region of the germline. Scale bars, 5μm.
