# Supplementary material for: Distinct regions of the intrinsically disordered protein MUT-16 mediate assembly of a small RNA amplification complex and promote phase separation of Mutator foci
Source: PLoS Genet. 2018 Jul 23;14(7):e1007542. doi: 10.1371/journal.pgen.1007542 (PMC6072111; doi:10.1371/journal.pgen.1007542)
Supplement: S3 Fig — (A) Deletion strain controls were assessed for RNAi response to somatic (nhr-23 and lin-29) or germline (pos-1) RNAi. For somatic RNAi, P0 animals were categorized as having fully penetrant (red bars) or intermediate RNAi defects (gray bars). Intermediate effects were categorized as non-thriving, slow moving adults with eggs for nhr-23 and as adults with protruding vulvas for lin-29. For pos-1, the F1 eggs and hatched larvae were counted to calculate % viable progeny from treated P0 animals. Weighted means and standard deviations were calculated from three independent RNAi trials of n = ~20 for nhr-23 and lin-29, and n = ~140 F1 eggs from 4 P0 adults for pos-1. (B) Deletion strains were assessed for RNAi response and categorized as having either fully penetrant RNAi defects (Rde, red boxes), intermediate RNAi defects (Weak, pink boxes), or wild-type response (WT, white boxes). ND indicates that strain was not constructed or scored. For nhr-23, fully penetrant RNAi defects are strains where >65% of animals are non-arrested and healthy, intermediate RNAi defects are strains where >35% are non-arrested (either healthy or sick), and wild-type response are strains where ≤35% are non-arrested (either healthy or sick). For lin-29, fully penetrant RNAi defects are strains where >70% of animals were scored as non-burst, intermediate RNAi defects are strains where 35%-70% of animals were scored as non-burst, and wild-type response are strains where <35% are non-burst. For pos-1, fully penetrant RNAi defects are strains where >75% F1s are viable, intermediate RNAi defects are strains where 10–75% F1 are viable, and wild-type response are strains <10% F1 are viable. (PDF) [file pgen.1007542.s003.pdf]

A

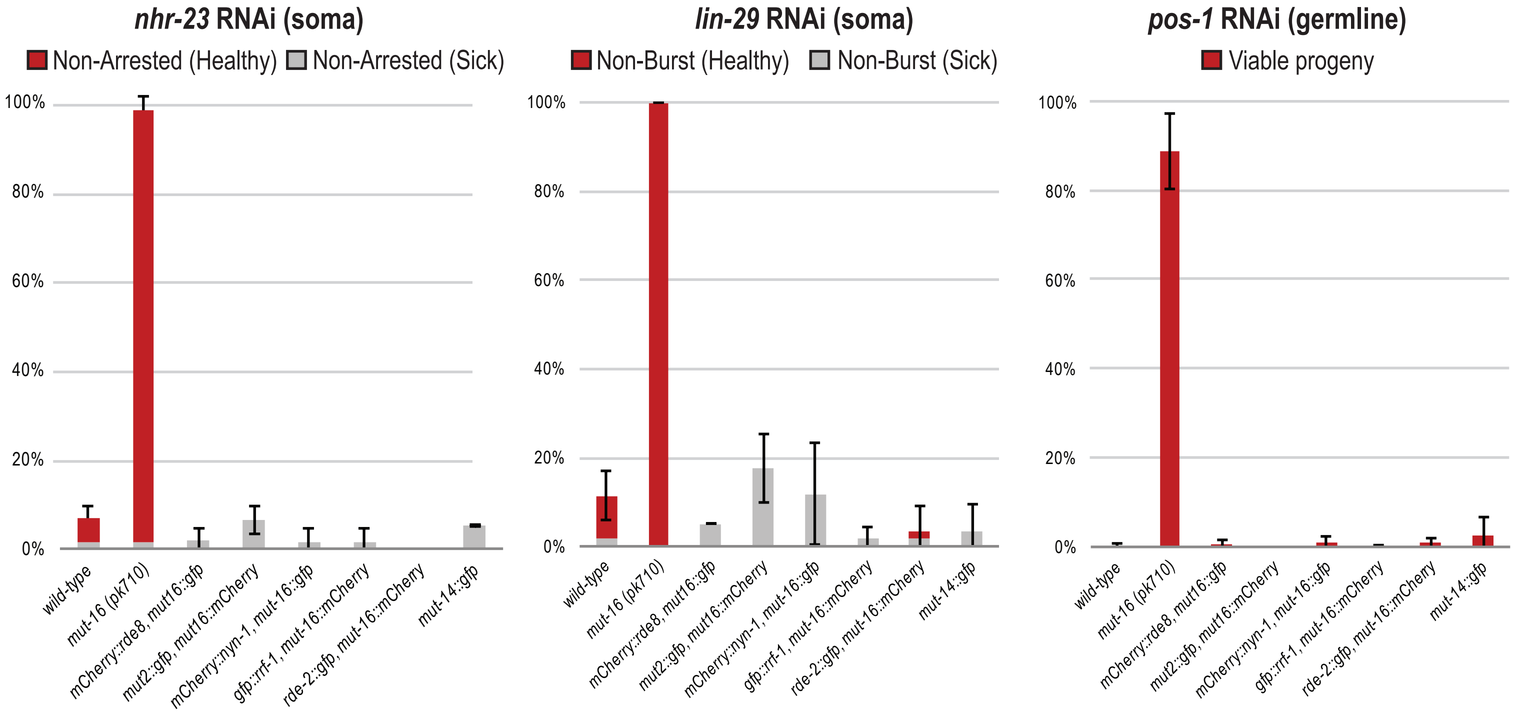

B

nhr-23

Deletion	*mut-2*	*rde-8*	*rrf-1*	*rde-2*	*mut-14*	*nyn-1*
ΔA	WT	WT	WT	WT	WT	ND
ΔB	Weak	Weak	Weak	Weak	Weak	Weak
ΔC	Rde	Rde	Rde	Rde	Rde	Rde
ΔD	Weak	Weak	Weak	Weak	Weak	Weak
ΔE	WT	WT	WT	WT	WT	ND
ΔF	Rde	Rde	Rde	Rde	Rde	ND
ΔG	WT	WT	WT	WT	Weak	ND
ΔH-I	Rde	Rde	Rde	Rde	Rde	ND
ΔJ	WT	WT	ND	ND	ND	ND
ΔK	WT	WT	ND	ND	ND	ND
ΔL	Rde	Rde	ND	ND	ND	ND

lin-29

Deletion	*mut-2*	*rde-8*	*rrf-1*	*rde-2*	*mut-14*	*nyn-1*
ΔA	WT	WT	WT	WT	WT	ND
ΔB	WT	WT	WT	WT	WT	WT
ΔC	Weak	Rde	Rde	Rde	Weak	Rde
ΔD	WT	WT	WT	WT	WT	WT
ΔE	WT	WT	WT	WT	WT	ND
ΔF	Rde	Rde	Rde	Rde	Rde	ND
ΔG	WT	WT	WT	WT	Weak	ND
ΔH-I	Rde	Rde	Rde	Weak	Rde	ND
ΔJ	WT	WT	ND	ND	ND	ND
ΔK	WT	WT	ND	ND	ND	ND
ΔL	Weak	Weak	ND	ND	ND	ND

pos-1

Deletion	*mut-2*	*rde-8*	*rrf-1*	*rde-2*	*mut-14*	*nyn-1*
ΔA	Weak	WT	Weak	WT	Weak	ND
ΔB	Rde	Rde	Rde	Rde	Rde	Rde
ΔC	Rde	Rde	Rde	Rde	Rde	Rde
ΔD	Rde	Rde	Rde	Rde	Rde	Rde
ΔE	Rde	Rde	Rde	Rde	Rde	ND
ΔF	Rde	Rde	Rde	Rde	Rde	ND
ΔG	Weak	Weak	WT	WT	Weak	ND
ΔH-I	Rde	Rde	Rde	Rde	Rde	ND
ΔJ	Rde	Rde	ND	ND	ND	ND
ΔK	Rde	Rde	ND	ND	ND	ND
ΔL	Rde	Rde	ND	ND	ND	ND

S3 Fig. Response of GFP- and mCherry-tagged strains to somatic and germline RNAi.

(A) Deletion strain controls were assessed for RNAi response to somatic (*nhr-23* and *lin-29*) or germline (*pos-1*) RNAi. For somatic RNAi, P0 animals were categorized as having fully penetrant (red bars) or intermediate RNAi defects (gray bars). Fully penetrant RNAi defects were larval arrest for *nhr-23*, vulval burst for *lin-29*, and non-hatching F1 for *pos-1*. Intermediate effects were categorized as non-thriving, slow moving adults with eggs for *nhr-23* and as adults with protruding vulvas for *lin-29*. For *pos-1*, the F1 eggs and hatched larvae were counted to calculate % viable progeny from treated P0 animals. Weighted means and standard deviations were calculated from three independent RNAi trials of n=~20 for *nhr-23* and *lin-29*, and n=~140 F1 eggs from 4 P0 adults for *pos-1*.

(B) Deletion strains were assessed for RNAi response and categorized as having either fully penetrant RNAi defects (Rde, red boxes), intermediate RNAi defects (Weak, pink boxes), or wild-type response (WT, white boxes). ND indicates that strain was not constructed or scored. For *nhr-23*, fully penetrant RNAi defects are strains where >65% of animals are non-arrested and healthy, intermediate RNAi defects are strains where >35% are non-arrested (either healthy or sick), and wild-type response are strains where ≤35% are non-arrested (either healthy or sick). For *lin-29*, fully penetrant RNAi defects are strains where >70% of animals were scored as non-burst, intermediate RNAi defects are strains where 35%-70% of animals were scored as non-burst, and wild-type response are strains where <35% are non-burst. For *pos-1*, fully penetrant RNAi defects are strains where >75% F1s are viable, intermediate RNAi defects are strains where 10-75% F1 are viable, and wild-type response are strains <10% F1 are viable.
