# Supplementary figures and images for: Distinct regions of the intrinsically disordered protein MUT-16 mediate assembly of a small RNA amplification complex and promote phase separation of Mutator foci
Source: PLoS Genet. 2018 Jul 23;14(7):e1007542. doi: 10.1371/journal.pgen.1007542 (PMC6072111; doi:10.1371/journal.pgen.1007542)

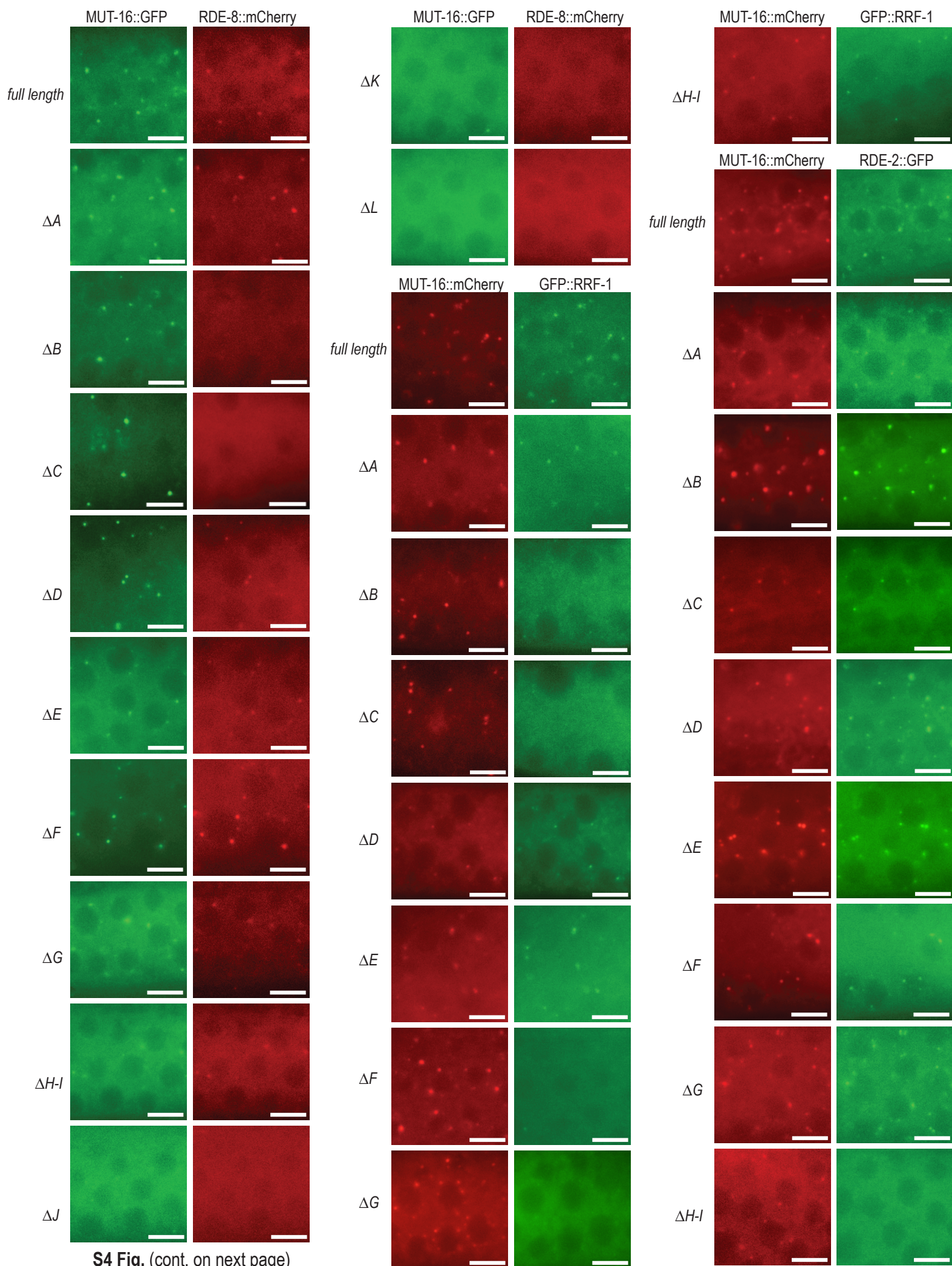

**S4 Fig. (cont. on next page)**

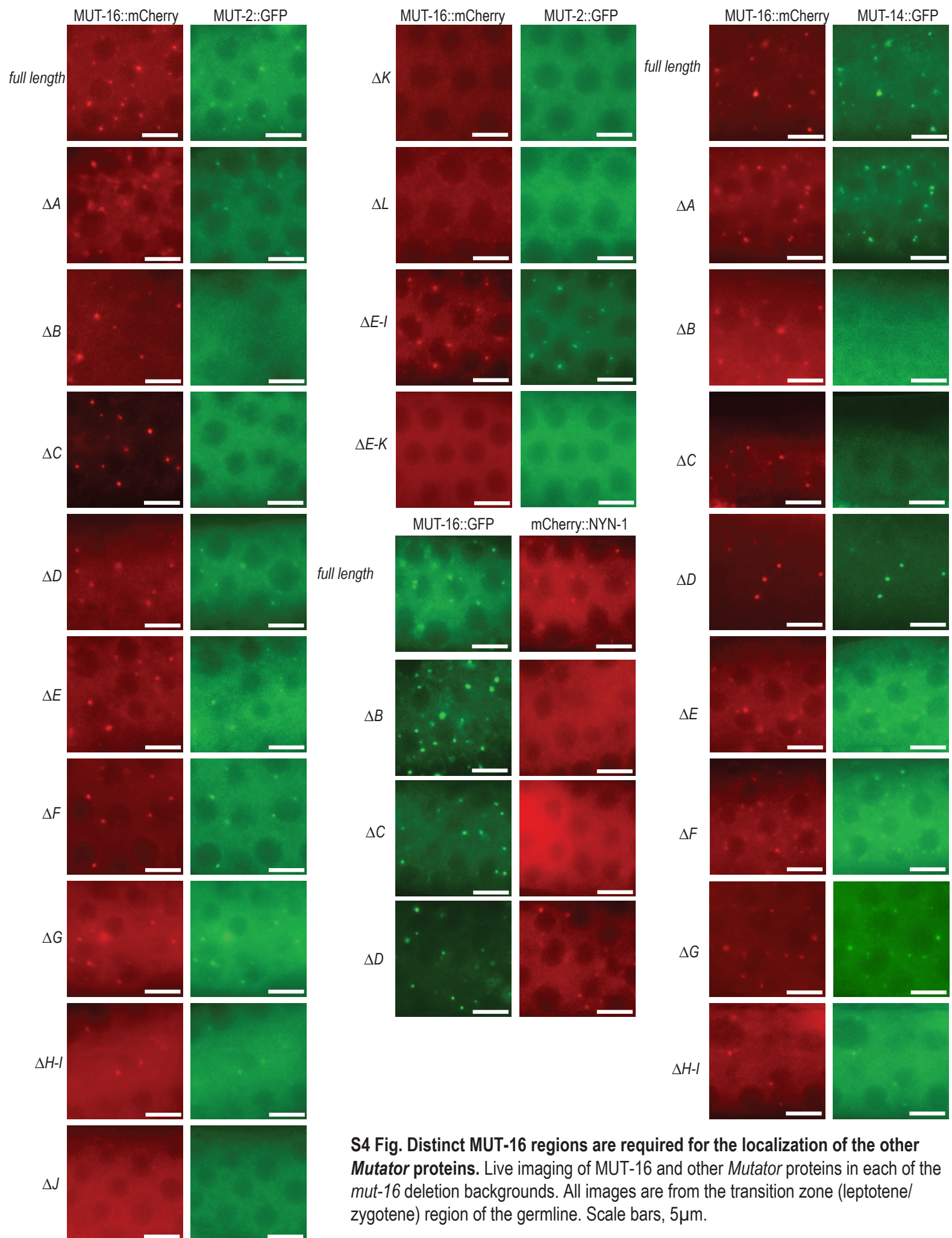

Supplement: S4 Fig — Live imaging of MUT-16 and other Mutator proteins in each of the mut-16 deletion backgrounds. All images are from the transition zone (leptotene/zygotene) region of the germline. Scale bars, 5μm. (PDF) [file pgen.1007542.s004.pdf]
