# Supplementary material for: Distinct regions of the intrinsically disordered protein MUT-16 mediate assembly of a small RNA amplification complex and promote phase separation of Mutator foci
Source: PLoS Genet. 2018 Jul 23;14(7):e1007542. doi: 10.1371/journal.pgen.1007542 (PMC6072111; doi:10.1371/journal.pgen.1007542)
Supplement: S5 Fig — Immunoprecipitation and western blot of MUT-16::mCherry::2xHA (expected sizes between 132–141 kD for MUT-16 deletions and 148 kD for MUT-16 full length) and MUT-2::GFP::3xFLAG (83 kD). Top three panels are total lysate from strains indicated above, and bottom two panels are following HA immunoprecipitation. Note that in some cases, full-length MUT-16 was degraded beyond the detection limit of the western blot in the input sample, but was still present following immunoprecipitation. The equivalent of ~0.5% of starting material for the input fractions and ~20% of starting material for the IP fractions were loaded onto the gels. (PDF) [file pgen.1007542.s005.pdf]

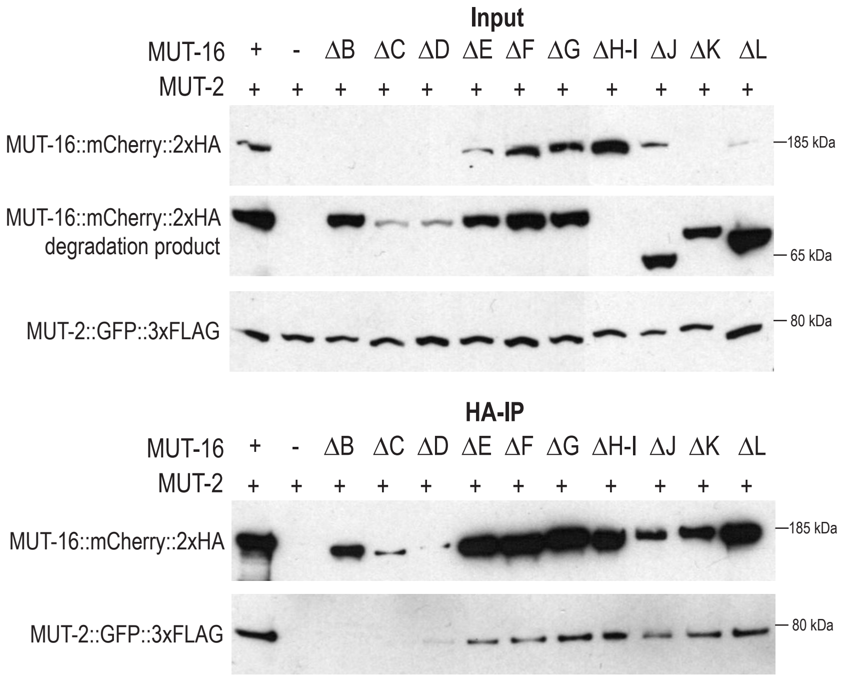

S5 Fig. Regions B and C of MUT-16 are required for interaction with MUT-2 *in vitro*.

Immunoprecipitation and western blot of MUT-16::mCherry::2xHA (expected sizes between 132 – 141 kD for MUT-16 deletions and 148 kD for MUT-16 full length) and MUT-2::GFP::3xFLAG (83 kD). Top three panels are total lysate from strains indicated above, and bottom two panels are following HA immunoprecipitation. Note that in some cases, full-length MUT-16 was degraded beyond the detection limit of the western blot in the input sample, but was still present following immunoprecipitation. The equivalent of ~0.5% of starting material for the input fractions and ~20% of starting material for the IP fractions were loaded onto the gels.
