# Supplementary material for: Distinct regions of the intrinsically disordered protein MUT-16 mediate assembly of a small RNA amplification complex and promote phase separation of Mutator foci
Source: PLoS Genet. 2018 Jul 23;14(7):e1007542. doi: 10.1371/journal.pgen.1007542 (PMC6072111; doi:10.1371/journal.pgen.1007542)
Supplement: S6 Fig — Representative images from the early pachytene region after L4 animals were subjected to 30°C heat shock for 6 hours and allowed to recover on plates at room temperature (~21°C) for the indicated amount of time. All images are from different animals to demonstrate the variability in foci presence and intensity at each time point. Scale bars, 5μm. (PDF) [file pgen.1007542.s006.pdf]

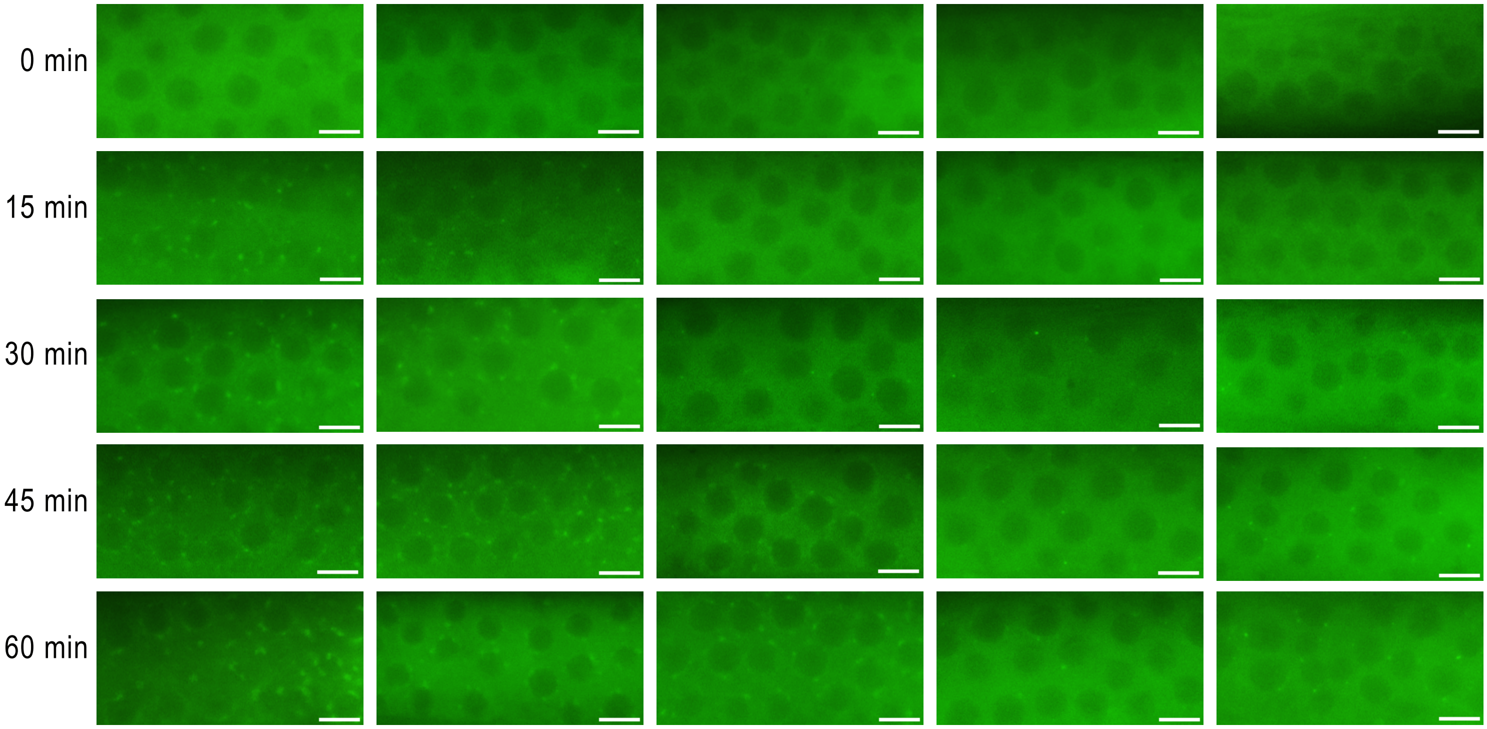

S6 Fig. MUT-16 foci are temperature dependent.
Representative images from the early pachytene region after L4 animals were subjected to 30°C heat shock for 6 hours and allowed to recover on plates at room temperature (~21°C) for the indicated amount of time. All images are from different animals to demonstrate the variability in foci presence and intensity at each time point. Scale bars, 5μm.
